# Performance of ionic liquid functionalized metal organic frameworks in the adsorption process of phenol derivatives

**DOI:** 10.1039/d3ra08024b

**Published:** 2024-02-05

**Authors:** Lavinia Lupa, Nick Samuel Tolea, Marcela Iosivoni, Bianca Maranescu, Nicoleta Plesu, Aurelia Visa

**Affiliations:** a Faculty of Industrial Chemistry and Environmental Engineering, Politehnica University Timisoara 6 Vasile Parvan Blv 300223 Timisoara Romania; b “Coriolan Dragulescu” Institute of Chemistry 24 Mihai Viteazul Blv 300223 Timisoara Romania apascariu@yahoo.com; c National Institute of Research and Development for Electrochemistry and Condensed Matter Dr. A. P. Podeanu 144 300569 Timişoara Romania; d Department of Biology-Chemistry, Faculty of Chemistry, Biology, Geography, West University 16 Pestalozzi Street 300115 Timisoara Romania

## Abstract

The growth of industrial activities has produced a significant increase in the release of toxic organic pollutants (OPs) to the environment from industrial wastewater. On this premise, this study reports the use of metal organic frameworks (MOFs) impregnated with various ionic liquids (ILs) in the adsorption of phenol derivatives, *i.e.*, 2,6-dimethylphenol and 4,4′-dihydroxybiphenyl. MOFs were prepared starting from 1-hydroxyethylidene-1,1-diphosphonic acid (HEDP) with divalent (Co, Ni, Cu) and trivalent (Ce) metal salts in mild hydrothermal conditions using water as a green solvent. Imidazolium base ionic liquids, namely 1-butyl-3-methylimidazolium trifluoromethanesulfonate, 1-butyl-3-methylimidazolium nitrate, 1-butyl-3-methylimidazolium chloride, and 1-hexyl-3-methyl-imidazolium chloride, were used to modify MOFs, leading to composite materials (IL@MOF), which show the structural characteristics of MOFs, and complement the advantages of ILs. SEM, EDX images, and TG data indicate that the IL is just attached on the surface of the adsorbent material, with no changes in crystal size or morphology, but with slightly altered thermal stabilities of IL@MOF composites compared to the original ILs and MOFs, pointing to some interionic interaction between IL and MOF. This research consists of equilibrium experiments, studying the effect of the initial concentration of OPs on the adsorption efficiency of the as-prepared MOFs and IL@MOF, in order to determine the influence of the nature of the adsorbent on its developed adsorption capacity and to investigate the performance of both ILs and MOFs. To determine the maximum adsorption capacity, several empirical isotherms were used: Langmuir, Freundlich, Redlich–Peterson, and Dubinin–Radushkevich. The characteristic parameters for each isotherm and the correlation coefficient (*R*^2^) were identified. The IL modification of MOFs increased the adsorption capacity of IL@MOF for the removal of phenol derivatives from aqueous solution. The adsorption capacity function of the MOF structure follows the trend CeHEDP > CoHEDP > NiHEDP > CuHEDP. The best performance was achieved by adsorbent materials based on Ce.

## Abstract 2

The growth of industrial activities has produced a significant increase in the release of toxic organic pollutants (OPs) to the environment from industrial wastewater.

## Graphical abstract

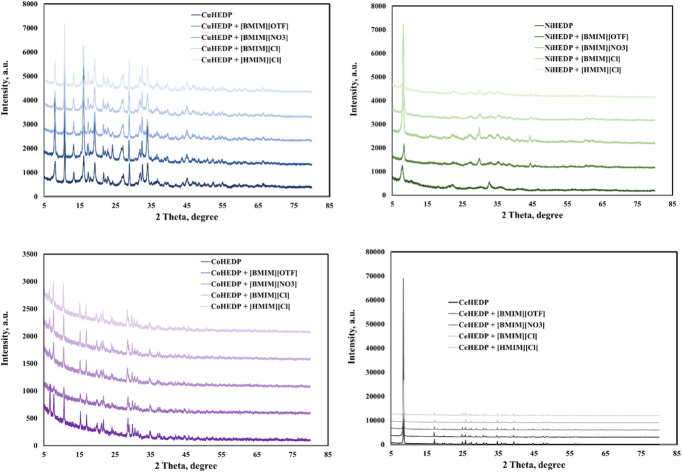

## 1. Introduction

Pollution management has become a severe environmental issue. Many industries produce phenol and phenol derivatives that end up in wastewater; these compounds are toxic and persistent even at very low concentration. Phenolic compounds are classified as one of the most toxic and carcinogenic elements to both living organisms and the environment.^1,2^ Thus, their removal from aqueous solution represents a continuing interest for researchers.^3^ Many studies have been carried out in this regard, and it has been proved that the adsorption process represents a viable solution, thanks to its efficiency, due to its ease of operation, low cost, and non-destructive nature.^4^ Another advantage of using the adsorption process for the removal of phenolic compounds from water is the multitude of existing adsorbents. Despite this large number, research is still focused on the continuing improvement of their adsorption performance.

Adsorption takes place through the uptake of pollutants (adsorbates) in most cases on the surface of the material (adsorbent) as a chemisorption process. Some reported adsorbents in this field include commercial activated carbon (AC),^5^ or a variety of biomass-derived adsorbents,^6,7^ zeolites,^8^ mesoporous silica,^9^ and graphene.^10^ Nevertheless, the majority of these materials have confronted the challenges of weak adsorption capacity, mild regeneration capability, and the production of toxic secondary products.^11^ The selection of innovative adsorbents with a wide range of applications is rarely straightforward.

The main properties required for adsorbent materials are simple synthesis, a large specific surface area for the material, and a thermally and chemically stable MOF.^12^ To achieve enhanced adsorption capacity, the surface of MOFs can be modified to increase surface energy, surface charge, roughness, hydrophobicity, and reactivity.^13^ Ionic liquids (ILs) are used for the selective adsorption of numerous pollutants, including dyes, drugs, phenolic compounds, and toxic heavy metal ions, which constitute significant risks to our environment, and ILs are therefore important to many industries. In the field of green chemistry, ILs are recognized as green solvents with the potential to replace traditional solvents and have been described as possibly environmentally beneficial in a range of applications. Ionic liquids have been extensively used in the liquid–liquid extraction of phenol compounds from aqueous solution. Their important roles in phenolic separation consist of the formation of hydrogen-bonding and hydrophobic interactions.^14,15^ But liquid–liquid extraction could only be applied for a solution containing a high concentration of phenolic compound and it implies the use of great quantities of ionic liquids, which increase the total cost of separation processes. Therefore, it is better to use ILs in a solid–liquid separation process, with their immobilisation on a suitable solid support.^16^ Many papers have investigated ILs immobilised on various solid supports and their activity in the adsorption process. In all cases, the adsorption capacities of the obtained adsorbent materials in the process of removal of the investigated pollutants increased, compared with the efficiency shown by unfunctionalized materials.^17–20^

For the goal of combining the advantages of both MOFs and IL_S_, IL-impregnated MOFs were synthesized for the adsorption of phenol derivatives. IL@MOF composites were previously studied for gas storage and separation,^21^ dye removal,^22,23^ or antibiotic adsorption.^24^ In each of these situations, MOFs and ILs work in concert, producing a synergistic effect, to increase the adsorption performance of the obtained materials for the purpose of removing the target pollutants. Nevertheless, there is still limited use of ILs impregnated on MOFs for investigation of various types of adsorption,^25^ and to the best of our knowledge the adsorption of phenolic derivatives using IL/MOF composites has not yet been studied.

In our previous study, we developed a highly selective phosphonate MOF for the removal of various heavy metals and rare earth elements from water,^26–28^ as well as a Zn–Al layered double hydroxide support impregnated with IL for pollutant removal.

From this premise, in this paper four different ionic liquids (ILs) were used to increase the functional groups on the surface of MOFs and thus enhance their adsorption abilities. The use of ILs allows an increase in functional groups on the MOF surface since their salts, which are found in a liquid state at room temperature, contain both an anion and a cation. Additionally, ILs have unique properties, such as negligible vapour pressure, low flammability, and chemical and thermal stability that render them green reagents/solvents.^29^ 1-Hydroxyethylidene-1,1-diphosphonic acid (HEDP), a bisphosphonate consisting of C–PO(OH)_2_ groups, was selected as the ligand in MOF preparation. The electron-rich groups of the HEDP hydroxyl and phosphonic acid groups have been demonstrated to have strong interactions with metal ions.^30–32^ In addition, HEDP has low toxicity to aquatic organisms, but causes eutrophication of water.^33^ The use of ILs on certain solid supports not only allows their advantages to be exploited, but it is also inexpensive because of the small quantities used.^17,18^

Their advantages are combined with the properties of MOFs, leading to improved adsorptive performance. In this paper, HEDP-type MOFs (Me-HEDP) containing four different cations – Cu^2+^, Ni^2+^, Co^2+^, and Ce^3+^ – were functionalized with four different imidazolium-based ILs: namely, 1-butyl-3-methylimidazolium trifluoromethanesulfonate, 1-butyl-3-methylimidazolium nitrate, 1-butyl-3-methylimidazolium chloride, and 1-hexyl-3-methyl-imidazolium chloride. Imidazolium-based ionic liquids were chosen for the impregnation of an Me-HEDP solid support because of their fine qualities compared with other ionic liquids, including high stability, high conductivity, facile dissolution in water due to dispersive interactions, low melting temperature, and a broad window of electrical stability. Their heteroatoms with a high nitrogen content lead to several chemical reactions which increase their reactivity and make imidazolium-based ionic liquids more suitable for adsorption processes.^16,34,35^ Besides these properties, imidazolium ionic liquids bring new functional groups to the obtained adsorbent materials. The first is the phosphonium group from the MOF solid support, and the second is the quaternary ammonium from the studied ionic liquids. In this case, adsorbent materials are obtained with multiple functional groups. From a literature survey it was concluded that ionic liquids from IL@MOF composites containing small alkyl chains present the highest thermal stability and develop the highest adsorption capacity.^36–38^ Therefore, ILs containing butyl and hexyl alkyl chains and the most studied anions – nitrate, chloride, and trifluoromethanesulfonate – were chosen.

The obtained materials were used as adsorbents for the removal of 2,6-dimethylphenol and 4,4′-dihydroxybiphenyl from aqueous solution to investigate the influence of the cation from the MOF structure and of the anion and alkyl chain from the ionic liquid on adsorption performance.

## 2. Experimental section

### 2.1 Materials

All chemicals of reagent grade quality were obtained from commercial sources and used without further purification. CoSO_4_·6H_2_O (50.0 mmol), Ce(NO_3_)_3_·6H_2_O (50.0 mmol), NiSO_4_·6H_2_O (50.0 mmol), and CuSO_4_·5H_2_O (50.0 mmol) were purchased from Merck (Milipore, Darmstadt, Germany). 1-Hydroxyethane-1,1-diphosphonic acid, sodium hydroxide, 1-butyl-3-methylimidazolium trifluoromethanesulfonate, 1-butyl-3-methylimidazolium nitrate, 1-butyl-3-methylimidazolium chloride, 1-hexyl-3-methyl-imidazolium chloride, 2,6-dimethylphenol, and 4,4′-dihydroxybiphenyl were obtained from Sigma-Aldrich Chemie GmbH (München, Germany), and urea from Alfa Aesar (Karlsruhe, Germany).

### 2.2 Synthesis of the MOF adsorbents

A quantity of 1-hydroxyethane-1,1-diphosphonic acid (60% aqueous solution, 100.0 mmol) was mixed with 100 mL of bi-distilled water, as a green solvent. To that solution, CoSO_4_·6H_2_O (50.0 mmol), Ce(NO_3_)_3_·6H_2_O (50.0 mmol), NiSO_4_·6H_2_O (50.0 mmol), or CuSO_4_·5H_2_O (50.0 mmol), and urea (50.0 mmol) were added under vigorous stirring. The pH was adjusted to 2.8–5 with an aqueous solution of NaOH (0.1 M). The solution was heated in an oil bath at 80 °C for 72 h, using milder reaction conditions of 80 °C compared to the 180 °C used in a previously described synthesis.^39^ The resulting crystals were collected by filtration, washed with bi-distilled water, and dried in air. The yield was calculated based on the metal salt: (yield: CoHEDP, [Co(HEDP)_2_(H_2_O)_2_]2H_2_O: 76%; NiHEDP, [Ni(HEDP)_2_(H_2_O)_2_]2H_2_O: 55%; CuHEDP, Cu(HEDP)_2_(H_2_O)_2_: 63%; CeHEDP, Ce(HEDP)(H_2_O)]3H_2_O: 69%).^26,39–43^

### 2.3 MOF functionalization with the studied ionic liquids

**Scheme 1:**
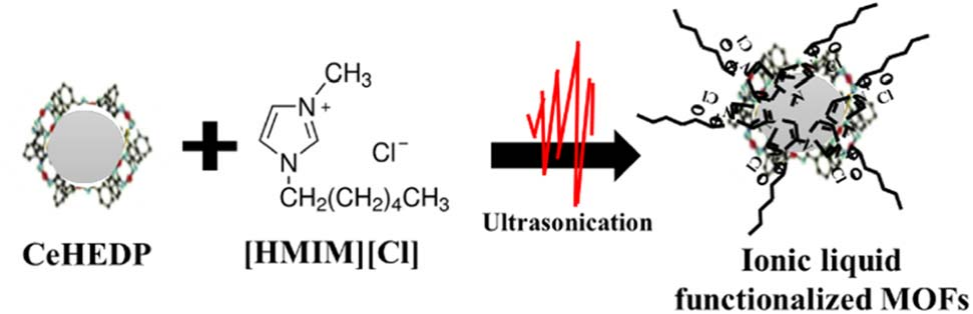
Ionic liquid functionalization of the studied MOFs through the ultrasonication method.

The impregnation of the synthesized MOFs with the studied ionic liquids was carried out by the ultrasound method, a rapid method that allows the uniform dispersion of the ionic liquids onto the surface of the MOFs (Scheme 1). The reaction between IL and MOF used for the functionalization process requires 10% of ionic liquid compared to the quantity of MOF materials. In the first step, the ionic liquid was dissolved in acetone using the ratio of IL to acetone of 0.1 g: 5 mL. The obtained suspension was sonicated for 10 minutes at room temperature under 100% amplitude using a Sonorex Super 10P ultrasonic bath. The suspension was then kept in an oven at 70 °C for 24 hours to evaporate the acetone. The symbols of the obtained and studied adsorbent materials are presented in Table 1.

**Table 1:** Abbreviations of the adsorbent materials used

No.	Symbol	Type of MOF	Type of IL
1	CuHEDP	CuHEDP	No ionic liquid
2	CuHEDP + [BMIM][OTF]		1-Butyl-3-methylimidazolium trifluoromethanesulfonate
3	CuHEDP + [BMIM][NO_3_]		1-Butyl-3-methylimidazolium nitrate
4	CuHEDP + [BMIM][Cl]		1-Butyl-3-methylimidazolium chloride
5	CuHEDP + [HMIM][Cl]		1-Hexyl-3-methyl-imidazolium chloride
6	NiHEDP	NiHEDP	No ionic liquid
7	NiHEDP + [BMIM][OTF]		1-Butyl-3-methylimidazolium trifluoromethanesulfonate
8	NiHEDP + [BMIM][NO_3_]		1-Butyl-3-methylimidazolium nitrate
9	NiHEDP + [BMIM][Cl]		1-Butyl-3-methylimidazolium chloride
10	NiHEDP + [HMIM][Cl]		1-Hexyl-3-methyl-imidazolium chloride
11	CoHEDP	CoHEDP	No ionic liquid
12	CoHEDP + [BMIM][OTF]		1-Butyl-3-methylimidazolium trifluoromethanesulfonate
13	CoHEDP + [BMIM][NO_3_]		1-Butyl-3-methylimidazolium nitrate
14	CoHEDP + [BMIM][Cl]		1-Butyl-3-methylimidazolium chloride
15	CoHEDP + [HMIM][Cl]		1-Hexyl-3-methyl-imidazolium chloride
16	CeHEDP	CeHEDP	No ionic liquid
17	CeHEDP + [BMIM][OTF]		1-Butyl-3-methylimidazolium trifluoromethanesulfonate
18	CeHEDP + [BMIM][NO_3_]		1-Butyl-3-methylimidazolium nitrate
19	CeHEDP + [BMIM][Cl]		1-Butyl-3-methylimidazolium chloride
20	CeHEDP + [HMIM][Cl]		1-Hexyl-3-methyl-imidazolium chloride

### 2.4 Analysis

The FTIR spectra were registered on a Jasco FT/IR-4200 instrument in the range 400–4000 cm^−1^ using the KBr pellet method. The TG-DTA information was recorded on an SDT-Q600 analyzer from TA Instruments. A PerkinElmer Diamond thermogravimetric analyzer was used, applying temperatures between 30 and 650 °C under a flow of air at a heating rate of 10 °C min^−1^. In order to identify the synthesized samples, X-ray diffraction (XRD) analysis was performed using a Rigaku Ultima IV X-ray diffractometer. For the pH measurement, an HI 2221 Calibration Check pH/ORP Meter from Hanna Instruments was used. SEM images were recorded using a Quanta FEG 250 microscope, equipped with an EDAX/ZAF quantifier. The adsorption studies were performed in batch mode, using for sample stirring a Julabo SW23 shaker bath at 200 rpm. A Varian Cary 50 spectrophotometer was used for adsorption studies.

### 2.5 Adsorption studies

The adsorption performance of the obtained materials was studied in the process of removing 2,6-dimethylphenol and 4,4′-dihydroxybiphenyl from aqueous solution. Equilibrium studies were carried out to determine the influence of cation type from the MOF structure and of the anion type and alkyl chain from the ionic liquid upon the adsorption performance.

The adsorption process took place in a Julabo SW23 shaker bath at 200 rpm constant speed for 1 h at room temperature 25 ± 2 °C, using an S : L ratio of 1 : 1 (0.025 g of adsorbent material and 25 mL of aqueous solution with the phenol compound). After the time had elapsed, the samples were filtrated, and the residual concentration of the studied phenolic compound in the resultant solutions was determined by UV-Vis spectrophotometry. In the 2,6-dimethylphenol adsorption process, an equilibration study was performed using solutions with concentrations of 10, 25, 50, 75, and 100 mg L^−1^. In the case of the 4,4′-dihydroxybiphenyl adsorption process, the used solutions contained concentrations of 10, 15, 20, 25, 30, and 40 mg L^−1^. For 4,4′-dihydroxybiphenyl, 40 mg L^−1^ was the highest solubility. The concentration of the phenolic compound before and after the adsorption process was measured at 269 nm for 2,6-dimethylphenol and at 262 nm for 4,4′-dihydroxybiphenyl. To ensure the reproducibility of the results, all the adsorption experiments were performed in triplicate, and the average values were used in data analysis. Relative standard deviations were found to be within ±0.8%.

The mass balance presented in eqn (1) was used to calculate the adsorption performance developed by the studied materials.1
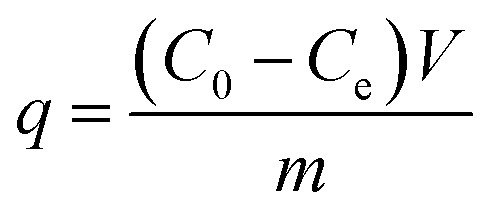
where *q* is the quantity of phenolic compound adsorbed onto the studied material (mg g^−1^); *C*_0_ and *C*_e_ represent the initial and equilibrium concentrations, respectively, of the phenolic compound in the solution (mg L^−1^); *V* is the volume of the solution (L); and *m* is the mass of the adsorbent (g) used in the experiments.

To determine the maximum adsorption capacity of the studied materials, to establish the influence of the nature of the adsorbent upon the developed adsorbent performance, the results obtained from the equilibrium studies were correlated with four adsorption isotherms: Langmuir, Freundlich, Redlich–Peterson, and Dubinin–Radushkevich.

Eqn (2) presents the linear form of the Langmuir isotherm. In this case, it is assumed that pollutant adsorption takes place upon a monolayer on the surface of the adsorbent material, where there is a uniform distribution of active sites:^27,44^2
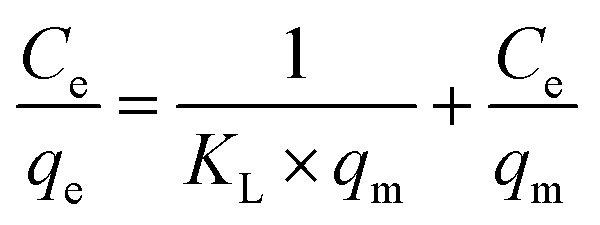
where *K*_L_ represents the Langmuir constant and *q*_m_ represents the maximum adsorption capacity developed by the functionalized MOFs. The *K*_L_ constant will be obtained from the slope of the linear plot of *C*_e_/*q*_e_*versus C*_e_.

The linear form of the Freundlich isotherm is presented in eqn (3). If the equilibrium data fit this isotherm, this means that adsorption takes place in a multiple layer due to the heterogeneous surfaces of the adsorbent material:^28,45^3
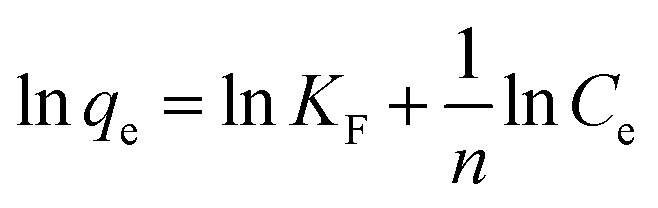
where *K*_F_ and 1/*n* represent the Freundlich parameters, which could be determined from the graphical representation of the ln *q*_e_ dependence function of ln *C*_e_.

The Redlich–Peterson isotherm incorporates the features of the Langmuir and Freundlich isotherms into a single equation (the linear form is presented in eqn (4)). A graphical representation of the function ln(*C*_e_/*q*_e_) = *f*(ln *C*_e_) enables the determination of the isotherm constants (*g* and *K*_R_):^46,47^4
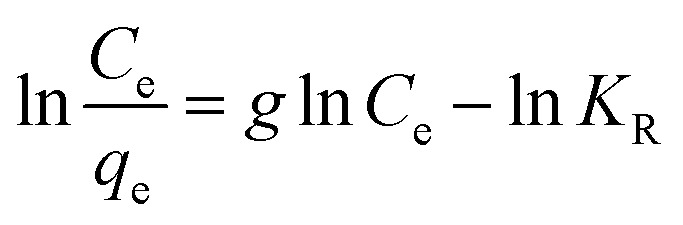


The design of the equilibrium data with the linear form of the Dubinin–Radushkevich isotherm (eqn (5)) allowed the sorption type (physical or chemical) to be established with the mean of the values obtained for the activation energy, *E* (eqn (6)):^48,49^

5 ln *q*_e_ = ln *q*_m_ − *K*_ad_ × *ε*^2^

where *K*_ad_ is the Dubinin–Radushkevich parameter and *ε* is the Polanyi adsorption potential:6
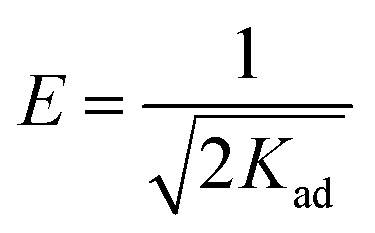


## 3. Results and discussion

### 3.1 Characterisation of adsorbent material

The adsorbents showed good crystallinity with well-defined peaks in the X-ray powder diffraction patterns, judging from the high intensity of the peaks in the 2*θ* region.

**Fig. 1:**
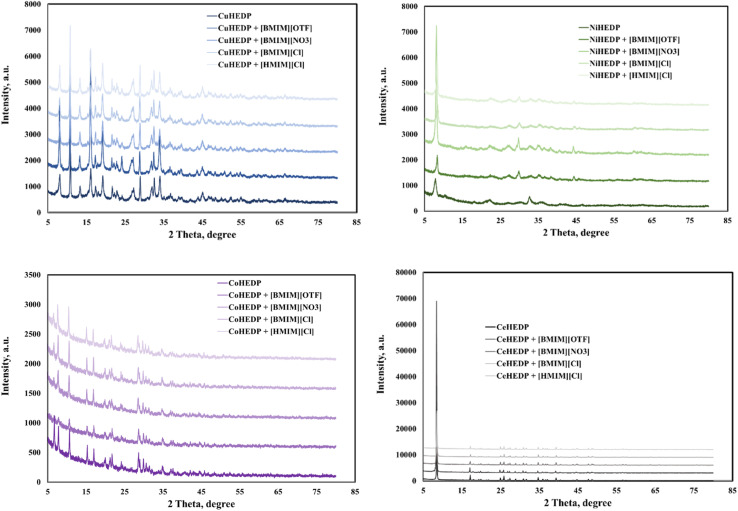
The XRD patterns of the studied adsorbent materials.

Fig. 1 shows that functionalization with the studied IL does not disturb the good structure of the studied MOFs; this indicates that the ionic liquid is just attached on the surface of the adsorbent material. In this manner, we will have an increased number of functional groups and therefore an increase in surface charge density. This is also supported by the SEM and EDX images, and TG.

No changes in crystal size or morphology have been observed for the investigated samples. The thermogravimetric analysis performed on the IL/MOF composites showed that the composites present altered thermal stabilities compared to the original ILs and MOFs, pointing to some interionic interaction between IL and MOF.^22^

Powder X-ray diffraction (PXRD) patterns are used in MOF characterization data to determine the phase purity and crystallinity of the material. CoHEDP and CoHEDP functionalized with ILs are amorphous, exhibit lower crystallinity, and have a baseline that is not flat compared with the other materials analyzed in this study.

**Fig. 2:**
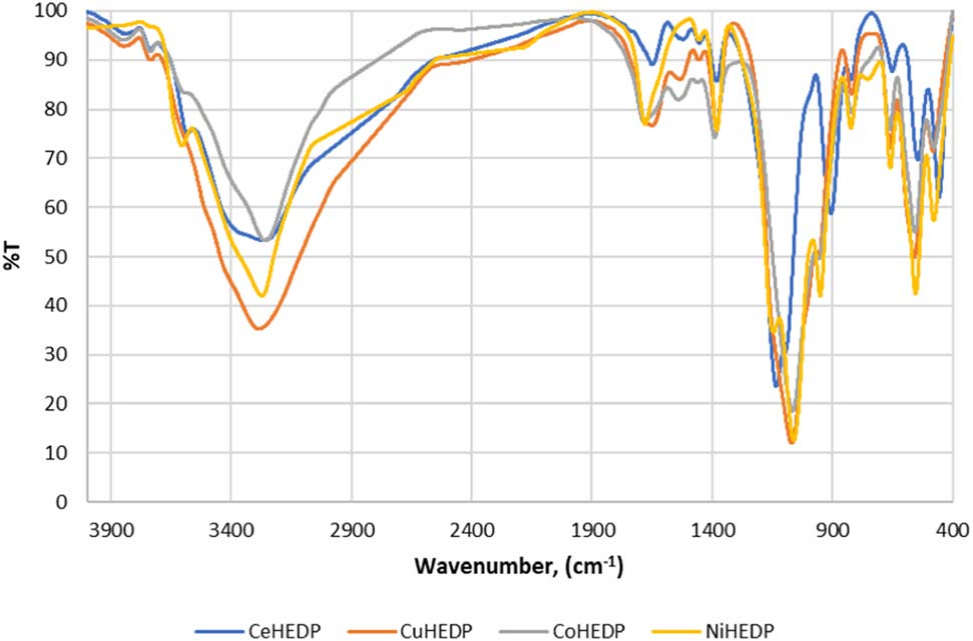
FTIR spectra for the adsorbent materials.

**Fig. 3:**
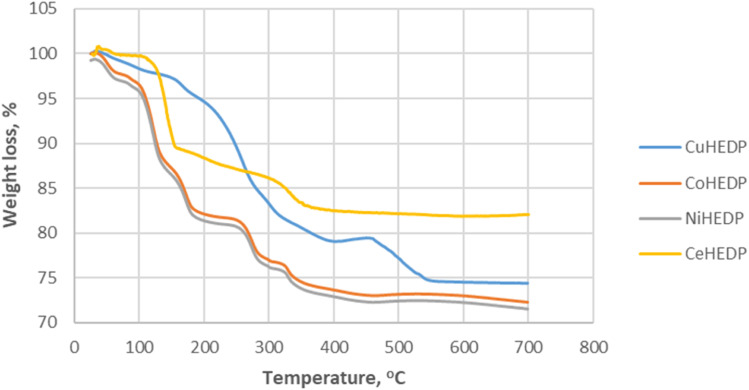
Thermal behavior of Me-HEDP in air, at a heating rate of 10 °C min^−1^.

In order to identify the formation of phosphonic metal organic networks, an FTIR spectroscopic investigation was performed (Fig. 2). Phosphonic acids have characteristic bands of the P–OH acid group in the range 2520–2700 cm^−1^. Thus, the first indication of the formation of the reaction product is the absence of P–O vibration from the acid groups. The range 900–1200 cm^−1^ is a complex spectral region characteristic of vibrations related to the –PO_3_ moiety of HEDP. In the FTIR spectra of the synthesized materials, a broad band was observed around 3400–3500 cm^−1^ corresponding to the water coordination molecules in the sample component (Fig. 2), which is also confirmed by the TGA diagrams (Fig. 3).

Thermogravimetric analysis of Me-HEDP compounds is revealed in Fig. 3, showing three main periods of weight loss. The humidity and solvent traces were removed from the adsorbents up to 105 °C. Accordingly, CuHEDP presents a 2.07% loss, NiHEDP a 1.96% loss, CeHEDP a 0.46% loss, and CoHEDP a 3.71% loss. In the temperature interval 110–250 °C, the second main weight loss corresponding to the loss of coordinated water molecules takes place. This is a two-stage process, as loss of water of crystallization proceeds progressively. A higher total weight of the coordinated water loss was observed in the case of CuHEDP (3.89%) and CoHEDP (13.58%) followed by Ce-HEDP (12.42%) and a lower loss in the case of NiHEDP (13.57%).

The third main mass loss takes place from 250 to 400 °C; all the adsorbents lose around 4.63–8.21% of their weight. This loss corresponds to the beginning of the decomposition of the MOFs. CuHEDP presents a 10.49% loss, NiHEDP an 8.21% loss, CeHEDP a 4.63% loss, and CoHEDP a 7.81% loss. The thermal decomposition continues in the case of Co-HEDP, Cu-HEDP, and NiHEDP above 400 °C that might be indicative of the formation of heat-stable organophosphide species between 300 and 400 °C. For example, similar TGA patterns have also been observed in the thermal decomposition profiles of a Cu-phenylphosphonic acid/porphyrin organic linker.^50,51^

### 3.2 Equilibrium studies

**Fig. 4:**
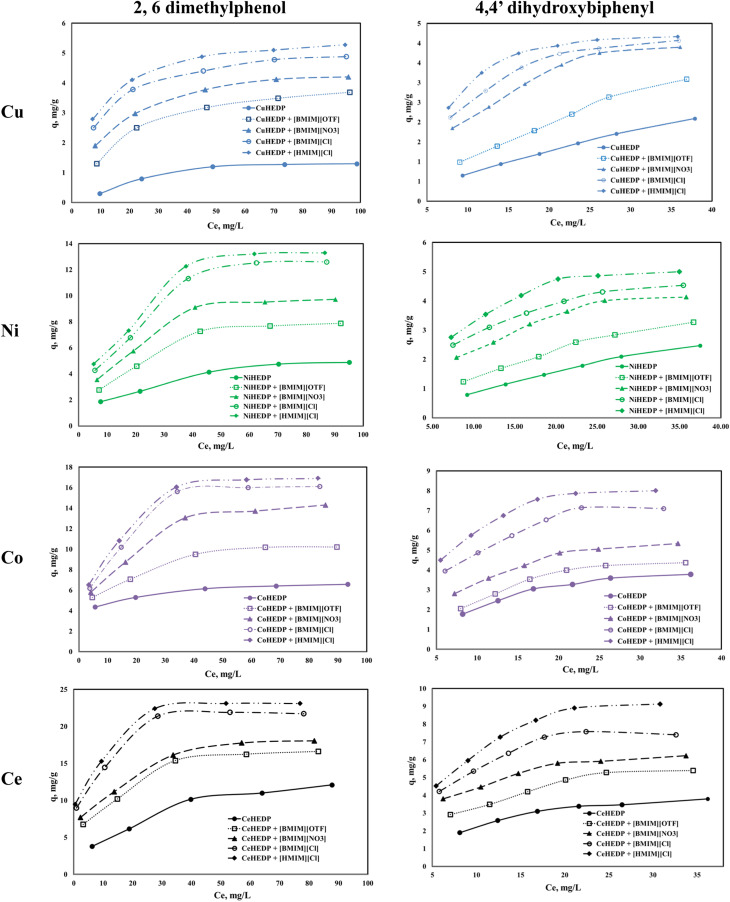
Equilibrium isotherm of adsorption of phenol compounds onto the studied materials.

The adsorption isotherms of the studied materials obtained in the process of removal of 2,6-dimethylphenol and of 4,4′-dihydroxybiphenyl are presented in Fig. 4.

It can be observed that for all the adsorbent materials and for both studied pollutants the adsorption capacity developed by the functionalized and unfunctionalized MOFs increase with increasing equilibrium concentration of the phenol compounds. The maximum adsorption capacities developed by the MOFs functionalized with the ILs are higher than those developed by the unfunctionalized samples. This means that the IL brings a greater benefit to the surface of the adsorbent materials due to the greater number of functional groups, which lead to better adsorption performance. Also, it could be observed that both the cation type from the MOF structure and the anion type from the ionic liquid influence the adsorption performance of the obtained material. The highest influence is observed in the case of the trivalent cation compared with the MOFs based on the divalent cation. This could be ascribed to the higher number of valence electrons available for electrostatic interaction.^52^

From the anion type of the studied ionic liquid, it was observed that the most efficient was the chloride anion. Its efficiency is higher if the alkyl chain is longer: 1-hexyl imidazolium chloride presents better adsorption capacity than 1-butyl imidazolium chloride. The increase in the alkyl chain from the ionic liquid leads to higher hydrophobicity of the obtained material.^53^

All the studied materials presented better performance in the process of removal of 2,6-dimethylphenol compared with the adsorption capacity developed in the process of removal of 4,4′-dihydroxybiphenyl. 4,4′-dihydroxybiphenyl presents a higher molar mass due to its two aromatic rings, which may slow the interaction between the active sites from the adsorbent surface and the OH groups from the pollutant.

The impregnation process can alter the density of the structures. Free rotation of the aromatic rings in the IL allows efficient π-stacking or edge-to-face interactions. The presence of a methyl group in IL somewhat stiffens the linker in a conformation where the aromatic rings are roughly orthogonal to the central core, which will reduce access to the adsorption sites. The hexyl group in IL brings about somewhat greater flexibility in the linker in a conformation in which the aromatic rings are oriented outwards and which leads to an increase in the number of adsorption sites.^54^ In this manner, the methyl imidazole-based ionic liquid blocks access to the active sites, whereas the hexyl imidazole-based ionic liquid has more access to the active sites. Since the structure is more open, the access of the pollutant will be facilitated.

**Fig. 5:**
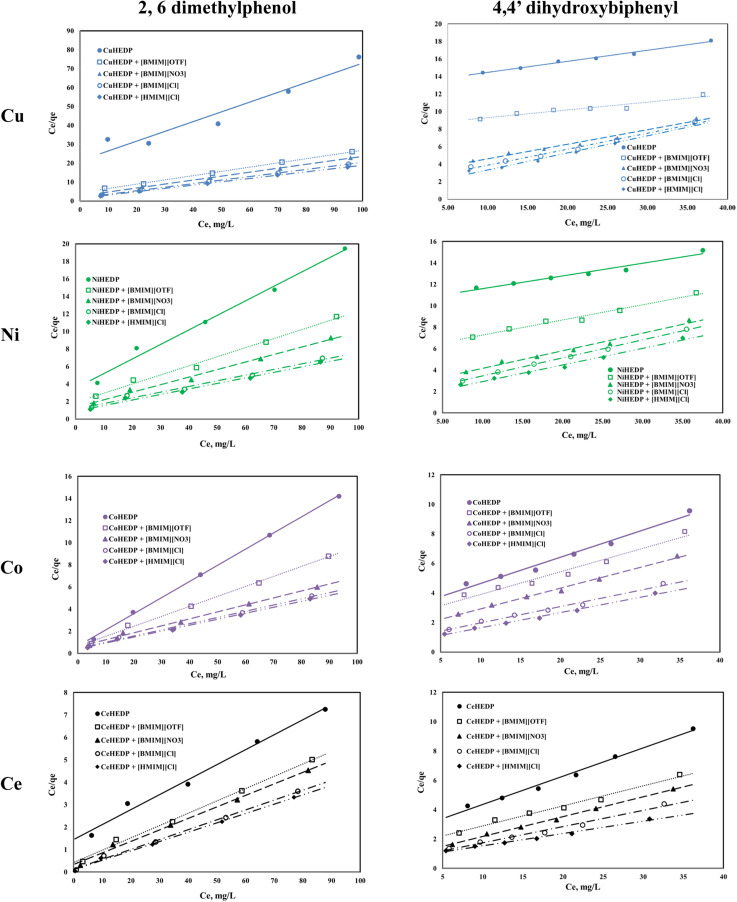
Langmuir isotherm of phenol compound adsorption onto the studied materials.

The graphical representation of the Langmuir isotherm for all the obtained adsorbent materials in the process of removal of 2,6-dimethylphenol and of 4,4′-dihydroxybiphenyl are presented in Fig. 5 and the obtained parameters of the Langmuir isotherm are summarized in Table 2.

**Table 2:** Langmuir isotherm parameters of phenol compound adsorption onto the studied materials

Adsorbent materials	2,6-Dimethylphenol				4,4′-Dihydroxybiphenyl			
	*q*_m_, exp, mg g^−1^	*q*_m_, calc, mg g^−1^	*K*_L_, L mg^−1^	*R* ^2^	*q*_m_, exp, mg g^−1^	*q*_m_, calc, mg g^−1^	*K*_L_, L mg^−1^	*R* ^2^
CuHEDP	1.29	1.94	0.0242	0.9830	2.09	7.96	9.45 × 10^−3^	0.9893
+ [BMIM][OTF]	3.69	4.43	0.0519	0.9985	3.09	11.21	0.0106	0.9215
+ [BMIM][NO_3_]	4.20	4.78	0.0797	0.9995	3.90	6.04	0.0559	0.9748
+ [BMIM][Cl]	4.88	5.33	0.114	0.9997	4.07	5.47	0.0888	0.9887
+ [HMIM][Cl]	5.28	5.70	0.128	0.9999	4.17	5.13	0.139	0.9874
NiHEDP	4.88	6.02	0.0474	0.9884	2.47	8.45	0.0114	0.9588
+ [BMIM][OTF]	7.87	9.61	0.0546	0.9910	3.27	7.08	0.0241	0.9780
+ [BMIM][NO_3_]	9.72	11.6	0.0653	0.9915	4.13	6.08	0.0651	0.9816
+ [BMIM][Cl]	12.59	15.4	0.0582	0.9854	4.53	5.93	0.0956	0.9976
+ [HMIM][Cl]	13.29	15.8	0.0693	0.9861	5.00	6.44	0.113	0.9883
CoHEDP	6.57	6.86	0.219	0.9992	3.78	5.62	0.0622	0.9802
+ [BMIM][OTF]	10.2	11.0	0.140	0.9968	4.37	6.53	0.0646	0.9653
+ [BMIM][NO_3_]	14.3	15.8	0.105	0.9952	5.33	7.10	0.0937	0.9931
+ [BMIM][Cl]	16.1	17.8	0.127	0.9948	7.10	9.03	0.128	0.9874
+ [HMIM][Cl]	16.9	18.6	0.137	0.9966	8.00	9.69	0.170	0.9940
CeHEDP	12.1	14.9	0.0464	0.9888	3.80	5.2	0.0787	0.9897
+ [BMIM][OTF]	16.6	18.2	0.129	0.9946	5.40	7.3	0.0899	0.9844
+ [BMIM][NO_3_]	18.06	19.5	0.151	0.9943	6.23	7.41	0.163	0.9971
+ [BMIM][Cl]	21.7	22.7	0.337	0.9963	7.40	9.07	0.169	0.9833
+ [HMIM][Cl]	23.1	23.9	0.395	0.9969	9.13	11.9	0.119	0.9905

The correlation coefficients obtained for the graphical representation of *C*_e_/*q*_e_*versus C*_e_ in the case of 2,6-dimethyilphenol adsorption onto the studied materials are greater than 0.99, with the exception of the MOFs based on Cu, Ni, and Ce without ionic liquid functionalisation. Also, in these cases, the maximum adsorption capacities obtained from the Langmuir representation are very close to those obtained experimentally. This means that 2,6-dimethylphenol is adsorbed as a monolayer onto the surface of the studied adsorbent due to the uniform distribution of the active sites on its surface.

In the case of 4,4′-dihydroxybiphenyl, much lower correlation coefficients were obtained, and the maximum adsorption capacities obtained from the Langmuir plots are much higher than those obtained experimentally. It is supposed that, due to the structure of dihydroxybiphenyl which contains two aromatic rings, the adsorption could not take place on a unique layer, but that on the contrary, there is an overlap, probably due to the hydrogen bonds formed by the hydroxyl groups in the structure.

The essential characteristics of the Langmuir isotherm can be expressed in terms of a dimensionless constant separation factor *R*_L_ that is given by eqn (7):7
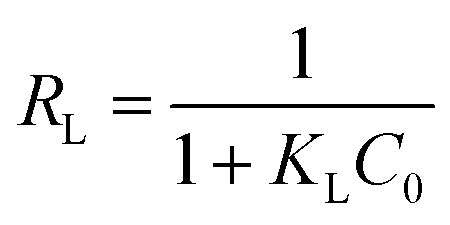
where *K*_L_ is the Langmuir constant and *C*_0_ is the initial concentration of phenol. The value of separation parameter *R*_L_ provides important information about the nature of adsorption. The value of *R*_L_ indicates the type of Langmuir isotherm: irreversible (*R*_L_ = 0), favourable (0 < *R*_L_ < 1), linear (*R*_L_ = 1), or unfavourable (*R*_L_ > 1).^19,44,55^*R*_L_ was found to be between 0 and 1 for the entire concentration range, and for all the studied materials, which indicates the favourable adsorption of phenol compounds onto the studied materials.

**Fig. 6:**
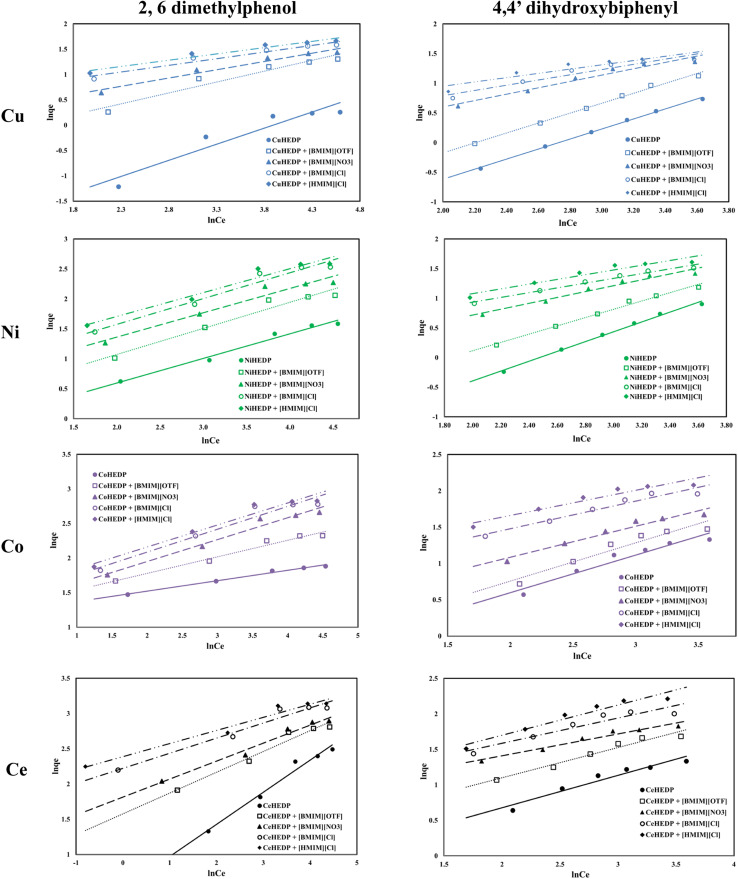
Freundlich isotherm of phenol compound adsorption onto the studied materials.

The graphical representation of the Freundlich isotherm for all the obtained adsorbent materials in the process of removal of 2,6-dimethylphenol and of 4,4′-dihydroxybiphenyl are presented in Fig. 6 and the obtained parameters are summarized in Table 3.

**Table 3:** Freundlich isotherm parameters of phenol compound adsorption onto the studied materials

Adsorbent materials	2,6-Dimethylphenol				4,4′-Dihydroxybiphenyl			
	*q*_m_, exp, mg g^−1^	1/*n*	*K*_F_, mg g^−1^	*R* ^2^	*q*_m_, exp, mg g^−1^	1/*n*	*K*_F_, mg g^−1^	*R* ^2^
CuHEDP	1.29	0.6368	0.084	0.9048	2.09	0.8453	0.0996	0.9980
+ [BMIM][OTF]	3.69	0.4284	0.57	0.9379	3.09	0.8362	0.158	0.9948
+ [BMIM][NO_3_]	4.20	0.3276	1.013	0.9633	3.90	0.5359	0.625	0.9586
+ [BMIM][Cl]	4.88	0.2632	1.56	0.9518	4.07	0.4385	0.919	0.9332
+ [HMIM][Cl]	5.28	0.2478	1.803	0.9524	4.17	0.3580	1.27	0.8628
NiHEDP	4.88	0.4102	0.797	0.9803	2.47	0.829	0.129	0.9944
+ [BMIM][OTF]	7.87	0.4347	1.23	0.9566	3.27	0.6996	0.277	0.9893
+ [BMIM][NO_3_]	9.72	0.4055	1.74	0.9542	4.13	0.4961	0.759	0.9621
+ [BMIM][Cl]	12.59	0.43	2.05	0.9619	4.53	0.4001	1.15	0.9778
+ [HMIM][Cl]	13.29	0.3983	2.49	0.9557	5.00	0.3975	1.33	0.9251
CoHEDP	6.57	0.1504	3.39	0.9898	3.78	0.5178	0.645	0.9399
+ [BMIM][OTF]	10.2	0.2398	3.66	0.9737	4.37	0.5258	0.746	0.9255
+ [BMIM][NO_3_]	14.3	0.3162	3.73	0.9709	5.33	0.4267	1.26	0.9568
+ [BMIM][Cl]	16.1	0.3305	4.16	0.9459	7.10	0.3796	2.05	0.9468
+ [HMIM][Cl]	16.9	0.3182	4.59	0.9556	8.00	0.3452	2.64	0.9291
CeHEDP	12.1	0.456	1.67	0.9782	3.80	0.4569	0.789	0.9364
+ [BMIM][OTF]	16.6	0.2946	4.84	0.9669	5.40	0.4264	1.28	0.9593
+ [BMIM][NO_3_]	18.06	0.2552	6.14	0.9768	6.23	0.3084	2.21	0.9603
+ [BMIM][Cl]	21.7	0.2139	9.23	0.9597	7.40	0.3548	2.41	0.8954
+ [HMIM][Cl]	23.1	0.1859	10.9	0.9663	9.13	0.4261	2.33	0.9446

Graphical representation of the obtained experimental data with the function ln *q*_e_ = *f*(ln *C*_e_) (Fig. 3) leads, with the exception of 4,4′-dihydroxybiphenyl adsorption, to low correlation coefficients, which indicates that the adsorption process could not be described by the Freundlich isotherm. Even in the cases where higher correlation coefficients were obtained, no good agreement could be observed between the experimentally obtained maximum adsorption capacity and the calculated value. One important characteristic of the Freundlich isotherm is the 1/*n* parameter, which is less than one for all the studied materials, also indicating the high affinity of the studied materials for the studied phenol compounds.^45^

**Fig. 7:**
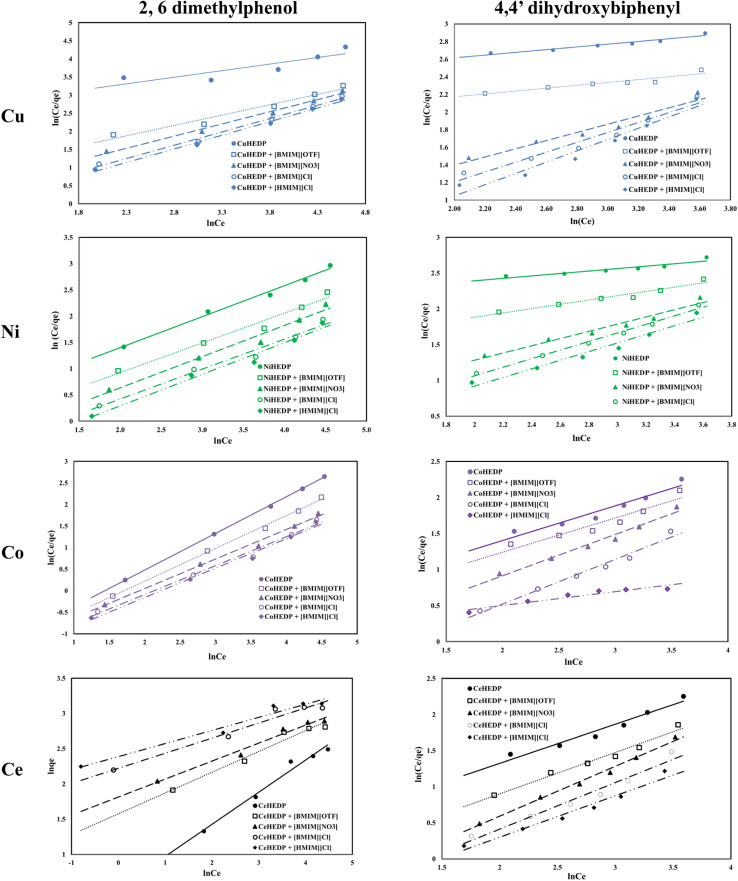
Redlich–Peterson isotherm of phenol compound adsorption onto the studied materials.

The Redlich–Peterson isotherms combine elements from both the Langmuir and Freundlich equations, if the adsorption mechanism corresponds to a hybrid process which follows a combination of monolayer and multilayer adsorption processes. The Redlich–Peterson graphical representations of phenol compound adsorption onto ionic liquid functionalized MOFs are presented in Fig. 7. The obtained parameters, together with the correlation coefficients, are listed in Table 4. The values of the correlation coefficients were found to be as good as those of the Langmuir isotherm model, especially in the process of removal of 2,6-dimethylphenol, which indicates that this isotherm model could also be applied for the design of the equilibrium sorption data of phenol compound removal using the studied materials. The close correspondence of the experimental data with both Langmuir and Redlich–Peterson isotherms confirms the fact that the adsorption mechanism of the studied phenol compounds onto ionic liquid functionalized MOFs is a hybrid adsorption process which involves an unideal monolayer sorption. Similar results have been reported by other researchers.^56^

**Table 4:** Redlich–Peterson isotherm parameters of phenol compound adsorption onto the studied materials

Adsorbent materials	2,6-Dimethylphenol				4,4′-Dihydroxybiphenyl			
	*q*_m_, exp, mg g^−1^	*g*, L mg^−1^	*K*_R_, L g^−1^	*R* ^2^	*q*_m_, exp, mg g^−1^	*g*, L mg^−1^	*K*_R_, L g^−1^	*R* ^2^
CuHEDP	1.29	0.3632	0.084	0.9555	2.09	0.1547	0.0996	0.9455
+ [BMIM][OTF]	3.69	0.5716	0.57	0.9641	3.09	0.1638	0.158	0.9491
+ [BMIM][NO_3_]	4.20	0.6724	1.013	0.9910	3.90	0.4641	0.625	0.9456
+ [BMIM][Cl]	4.88	0.7368	1.56	0.9936	4.07	0.5615	0.919	0.9582
+ [HMIM][Cl]	5.28	0.7522	1.803	0.9946	4.17	0.642	1.27	0.9529
NiHEDP	4.88	0.5898	0.797	0.9904	2.47	0.171	0.129	0.9424
+ [BMIM][OTF]	7.87	0.5653	1.23	0.9739	3.27	0.3004	0.277	0.9444
+ [BMIM][NO_3_]	9.72	0.5945	1.74	0.9782	4.13	0.5039	0.759	0.9632
+ [BMIM][Cl]	12.59	0.57	2.05	0.9780	4.53	0.5999	1.15	0.9900
+ [HMIM][Cl]	13.29	0.6017	2.49	0.9801	5.00	0.6025	1.33	0.9659
CoHEDP	6.57	0.8496	3.39	0.9997	3.78	0.4822	0.645	0.9714
+ [BMIM][OTF]	10.2	0.7602	3.66	0.9973	4.37	0.4742	0.746	0.9600
+ [BMIM][NO_3_]	14.3	0.6838	3.73	0.9936	5.33	0.5733	1.26	0.9756
+ [BMIM][Cl]	16.1	0.6695	4.16	0.9863	7.10	0.6204	2.05	0.9794
+ [HMIM][Cl]	16.9	0.6818	4.59	0.9900	8.00	0.1919	2.64	0.9636
CeHEDP	12.1	0.544	1.67	0.9846	3.80	0.5431	0.789	0.9741
+ [BMIM][OTF]	16.6	0.7054	4.84	0.9941	5.40	0.5736	1.28	0.9771
+ [BMIM][NO_3_]	18.06	0.7448	6.14	0.9972	6.23	0.6916	2.21	0.9918
+ [BMIM][Cl]	21.7	0.7861	9.23	0.9969	7.40	0.6452	2.41	0.9659
+ [HMIM][Cl]	23.1	0.8141	10.9	0.9982	9.13	0.5739	2.33	0.9687

The values obtained for the *g* parameter (Table 4) lie between 0 and 1, for all the studied adsorbents, indicating, once again, the favorable adsorption of phenol compounds onto the studied materials.

**Fig. 8:**
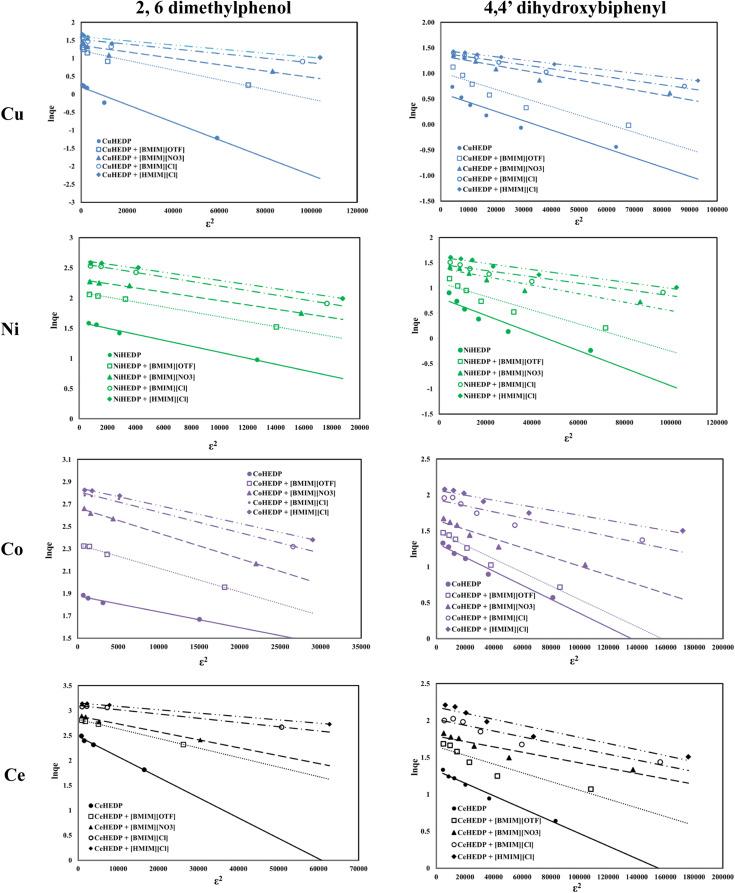
Dubinin–Radushkevich isotherm of phenol compound adsorption onto the studied materials.

The fitting of the experimental data with the Dubinin–Radushkevich model for all the studied adsorbent materials in the process of removal of 2,6-dimethylphenol and of 4,4′-dihydroxybiphenyl are presented in Fig. 8 and the obtained parameters together with the resulting correlation coefficients are listed in Table 5.

**Table 5:** Dubinin–Radushkevich isotherm parameters of phenol compound adsorption onto the studied materials

Adsorbent materials	2,6-Dimethylphenol				4,4′-Dihydroxybiphenyl			
	*q*_m_, exp, mg g^−1^	*K*_ad_, mol^2^ J^−2^	*E*, kJ mol^−1^	*R* ^2^	*q*_m_, exp, mg g^−1^	*K*_ad_, mol^2^ J^−2^	*E*, kJ mol^−1^	*R* ^2^
CuHEDP	1.29	2 × 10^−5^	0.16	0.9701	2.09	2 × 10^−5^	0.16	0.8939
+ [BMIM][OTF]	3.69	1 × 10^−5^	0.22	0.9544	3.09	2 × 10^−5^	0.16	0.885
+ [BMIM][NO_3_]	4.20	9 × 10^−6^	0.24	0.9147	3.90	1 × 10^−5^	0.22	0.9211
+ [BMIM][Cl]	4.88	6 × 10^−6^	0.29	0.9376	4.07	8 × 10^−6^	0.25	0.9684
+ [HMIM][Cl]	5.28	6 × 10^−6^	0.29	0.9319	4.17	6 × 10^−6^	0.29	0.9989
NiHEDP	4.88	5 × 10^−5^	0.10	0.9905	2.47	2 × 10^−5^	0.16	0.9061
+ [BMIM][OTF]	7.87	4 × 10^−5^	0.11	0.9976	3.27	1 × 10^−5^	0.22	0.9032
+ [BMIM][NO_3_]	9.72	4 × 10^−5^	0.11	0.9960	4.13	8 × 10^−6^	0.25	0.9074
+ [BMIM][Cl]	12.59	4 × 10^−5^	0.11	0.9990	4.53	6 × 10^−6^	0.29	0.9557
+ [HMIM][Cl]	13.29	3 × 10^−5^	0.13	0.9976	5.00	6 × 10^−6^	0.29	0.9185
CoHEDP	6.57	1 × 10^−5^	0.22	0.9812	3.78	1 × 10^−5^	0.22	0.9711
+ [BMIM][OTF]	10.2	2 × 10^−5^	0.16	0.9973	4.37	9 × 10^−6^	0.24	0.9638
+ [BMIM][NO_3_]	14.3	2 × 10^−5^	0.16	0.9980	5.33	6 × 10^−6^	0.29	0.9358
+ [BMIM][Cl]	16.1	2 × 10^−5^	0.16	0.9918	7.10	4 × 10^−6^	0.34	0.8888
+ [HMIM][Cl]	16.9	2 × 10^−5^	0.16	0.9987	8.00	3 × 10^−6^	0.41	0.9420
CeHEDP	12.1	4 × 10^−5^	0.11	0.9907	3.80	9 × 10^−6^	0.24	0.977
+ [BMIM][OTF]	16.6	2 × 10^−5^	0.16	0.9998	5.40	6 × 10^−6^	0.29	0.8642
+ [BMIM][NO_3_]	18.06	2 × 10^−5^	0.16	0.9917	6.23	4 × 10^−6^	0.34	0.8904
+ [BMIM][Cl]	21.7	8 × 10^−6^	0.25	0.9939	7.40	4 × 10^−6^	0.34	0.9287
+ [HMIM][Cl]	23.1	7 × 10^−6^	0.27	0.9989	9.13	4 × 10^−6^	0.34	0.9296

The equilibrium data applied to the Dubinin–Radushkevich model exhibit great correlation coefficients for the process of removal of 2,6-dimethylphenol. In all cases, the maximum adsorption capacities calculated from the Dubinin–Radushkevich model are very close to those obtained experimentally. The activation energies calculated in all cases were found to be less than 1, indicating that the adsorption of phenol compounds onto IL-functionalized MOFs corresponds to physical sorption, which involves electrostatic interaction and hydrogen bonds between the functional groups from the adsorbent surface and hydroxyl groups from the pollutant structure.

**Fig. 9:**
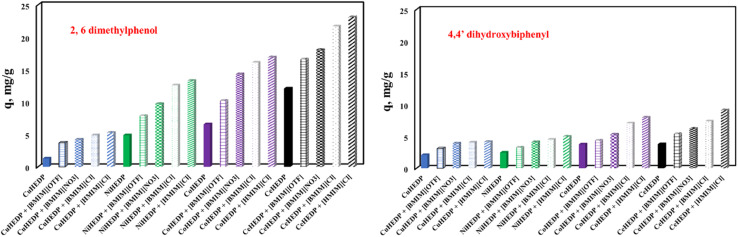
The influence of the nature of the adsorbent upon the adsorption performance developed in the process of removal of 2,6-dimethylphenol and 4,4′-dihydroxybiphenyl.

The influence of the nature of the adsorbent upon adsorption performance developed in the process of removal of 2,6-dimethylphenol and 4,4′-dihydroxybiphenyl could be observed from the plot of the maximum adsorption capacity obtained experimentally in each case, as presented in Fig. 9.

From the data reported in Fig. 9, it could be concluded that the nature of the adsorbent has a great influence upon the adsorption performance developed in the process of removal of phenol compounds from aqueous solution. The studied adsorbents present a much higher affinity for 2,6-dimethylphenol compared with the adsorption capacity developed for the removal of 4,4′-dihydroxybiphenyl. It was observed that the structure of the MOFs also influences the developed adsorption capacity. The adsorption capacity function of the MOF structure increases in the order CeHEDP > CoHEDP > NiHEDP > CuHEDP. The highest performance was achieved by the adsorbent materials based on Ce.

The MOFs functionalized with the investigated ionic liquid exhibited a greater maximum adsorption capacity than the unfunctionalized samples. This indicates that, because of the functional groups of the ionic liquid, the surface of the adsorbent material receives a greater number of actives sites, which improves the adsorption ability.^57–60^ Additionally, environmental research routinely uses computational methods to help experiments in order to speed them up and save expenses and resources.^61^

The adsorption capacity function of the anion from the ionic liquid structure increases in the order Cl^−^ > NO_3_^−^ > OTF^−^, indicating that the Cl^−^ anions lead to the highest surface charge density of the adsorbent surface.^62^

Longer alkyl chains result in increased chloride efficiency: 1-hexyl imidazolium chloride has greater adsorption capabilities than 1-butyl imidazolium chloride. The expanding alkyl chain of the ionic liquid caused the obtained substance to be more hydrophobic, leading to higher affinity for the studied pollutant.

**Fig. 10:**
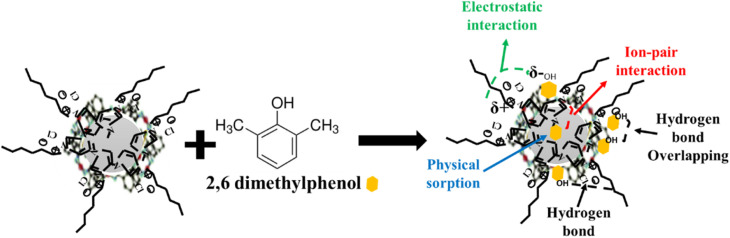
Mechanism of adsorption of 2,6-dimethylphenol onto CeHEDP + [HMIM][Cl].

Correlating the results of the characterisation section with those of equilibrium adsorption, a proposed mechanism for the adsorption of 2,6-dimethylphenol onto CeHEDP+[HMIM][Cl] – the material which showed the highest performance – is presented in Fig. 10.

Due to the small value obtained for the activation energy, it is obvious that the adsorption of 2,6-dimethylphenol onto the studied materials corresponds to a physical process.

**Fig. 11:**
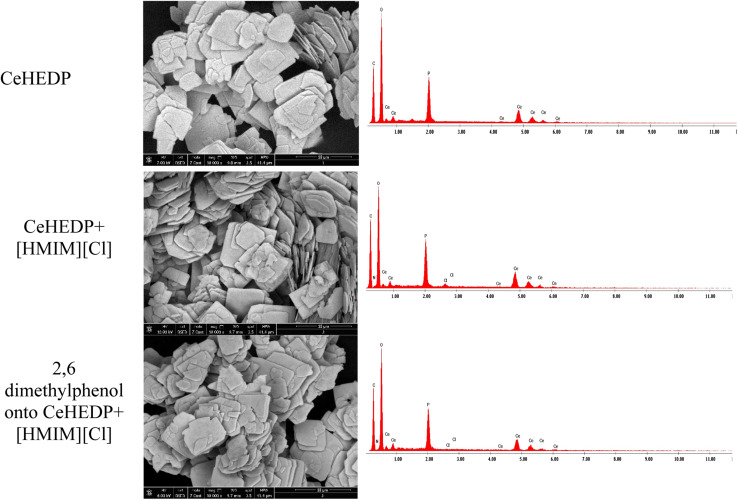
SEM and EDX images for CeHEDP, CeHEDP + [HMIM][Cl], and 2,6-dimethylphenol onto CeHEDP + [HMIM][Cl].

This physical process involves the following steps: (1) there is physical sorption into the cavities from the surface of the adsorbent obtained after ionic liquid functionalisation. From the SEM images (Fig. 11), it can be observed that the MOF surface is not very smooth; it becomes heterogenous after the functionalisation process. (2) There are electrostatic interactions between the positive charge of the imidazolium cation from the studied ionic liquids and the electronegativity of oxygen molecules from the pollutant. (3) There is ion-pair interaction between the electron pairs of the oxygen molecules from the pollutants and the opposite charge from the adsorbent surface. This could be the reason why Ce-based adsorbent materials developed the highest adsorption capacity: being a trivalent metal, Ce has higher charge density on its ion, compared with the studied divalent metals; therefore, it will interact strongly with an ion of the opposite charge. (4) There is a hydrogen bond between the H from the hydroxyl group from the pollutant and the H from the adsorbent materials. Also, this hydrogen bond leads to overlapping of the pollutant molecules onto the surface of the adsorbent, a fact confirmed by the good fit of the adsorption experimental data with the Redlich–Peterson isotherm model, which confirms that the adsorption process does not take place by ideal monolayer sorption.

The best adsorbent materials, CeHEDP and CeHEDP + [HMIM][Cl], were further investigated by SEM, EDX, and TG. From the SEM images (Fig. 11), it can be observed that the MOF surface is not very smooth; it becomes heterogenous after the functionalisation process.

In the case of CeHEDP, the humidity and solvent trace were eliminated from the adsorbents up to 105 °C, while in the case of CeHEDP-IL, the removal was 0.89% of the mass. The loss of coordinated water molecules accounts for the second major weight loss in the temperature range 110–250 °C. This is a two-step process where molecules of the water of crystallization are gradually released. The percentages were 13.56% for CeHEDP-IL and 12.42% for CeHEDP. The loss coincides with the onset of breakdown of the composite between 250 and 400 °C. In this temperature domain, CeHEDP shows a loss of 4.63%, while CeHEDP-IL shows a loss of 12.74%.

**Fig. 12:**
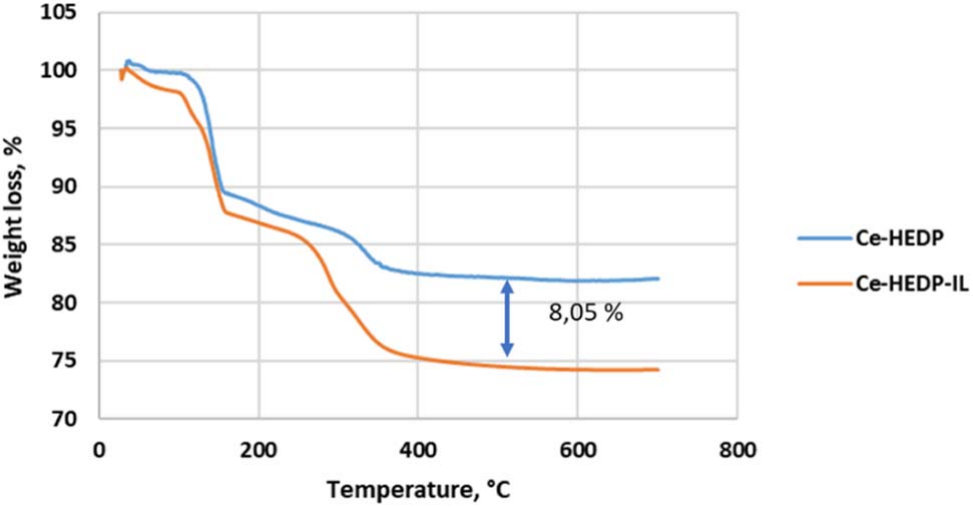
Thermal behavior of CeHEDP and CeHEDP + [HMIM][Cl] in air, at a heating rate of 10 °C min^−1^.

The TG shapes of CeHEDP before and after impregantion with IL are similar. Looking at the TG spectra, it was observed that the difference at 450 °C between CeHEDP and CeHEDP + [HMIM][Cl] is about 8.05% of the mass loss, close to the amount of IL impregnated on the surface (Fig. 12).

Thermogravimetric analysis performed on the IL@MOF composites showed that most of the composites present lower thermal stabilities compared to the original ILs and MOFs. IL@MOF composites with a functional group in their anions showed higher thermal stability limits than the ILs. The onset temperatures of the composites seem to be influenced by the nature and length of the alkyl chain, by the degree of functionalization of the cation and the nature of the anion present in the IL structure, and by the nature of the MOF.^63,64^ The interionic interaction directly controls the electronic environment over the metal sites. A stronger interaction between the IL and the metal sites will strongly affect the thermal stability. It was reported that the interactions between the IL and open Cu sites from CuBTC, one of the most widely studied MOFs for IL incorporation, are higher for an IL with a long alkyl chain.^65^

Our results reveal similar *T*_onset_ temperatures for Ce-HEDP and Ce HEDP-IL (where IL is 1-hexyl-3-methyl-imidazolium chloride) (see Fig. 12). Similar findings were seen for composite sorbents made using the direct mixing technique. In the case of MOF-177 impregnation with 1-ethyl-3-methylimidazolium acetate ([emim][Ac]), for [emim][Ac]@MOF-177-DI-10% an approximate 8% weight loss related to decomposition of the impregnated IL sample was reported.^66^ The shape of the TG curve obtained for Ce-HEDP and the impregnated compound is similar to those reported in the literature for CuBTC (Basolite C300) impregnated with 1,3-diethoxyimidazolium bis(trifluoromethylsulfonyl)imide [(EtO)_2_IM][NTf2], 1,3-dimethoxy-2-methylimidazolium bis(trifluoromethylsulfonyl)imide [(MeO)_2_MIM][NTf_2_], 1-butyl-3-methylimidazolium methyl sulfate [BMIM][MeSO_4_], and 1-butyl-3-methylimidazolium hexafluoroantimonate [BMIM][SbF_6_].^67^

The extremely versatile nature of IL@MOF composites and the remaining gaps in knowledge (*i.e.*, synthesis and uses) provide a wide range of opportunities for further discovery. In recent years, there has been an increasing amount of research conducted on the synthesis, characterization, and use of IL/MOF composites, making them quite modern components. Because of the opportunities created by combining the beneficial qualities of MOFs and ILs, IL/MOF hybrids offer a great deal of potential in many different fields. A recent study^68^ found that when these two elements are combined, IL@MOF composites perform better than pure MOFs in a wide range of applications, including gas storage, membrane-based gas separation, adsorption, ionic conductivity, catalysis, and superior selectivity and stability. To expand the scope of MOFs, one of the objectives is to rationalize materials in order to maximize their absorption capacity (or selectivity). Experimental data analysis (in special high-yield synthesis and testing) should be combined with molecular simulation in order to accelerate the discovery of relevant MOF structures.

## 4. Conclusions

In the present paper, the effects of the adsorbent nature of IL@MOF composites upon the efficiency of the process of removal of phenol derivatives (2,6-dimethylphenol and 4,4′-dihydroxybiphenyl) from aqueous solution have been investigated. It was observed that both the cation of the studied MOFs and the anion and the alkyl chain from the studied ionic liquids used for adsorbent functionalization influence the adsorption performance of the resulting materials.

From the experimental data, it is possible to conclude that the adsorption capacity developed by the studied materials decreases in the following order: CeHEDP > CoHEDP > NiHEDP > CuHEDP, both for the removal of 2,6-dimethylphenol and also for the removal of 4,4′-dihydroxybiphenyl. The adsorption capacity function of the anion from the ionic liquid structure increases in the order Cl^−^ > NO_3_^−^ > OTF^−^, with a maximum adsorption value of 23.1 mg g^−1^. Efficient π-stacking or edge-to-face interactions is made possible by the free rotation of aromatic rings in the interlayer gap. Therefore, access to the active sites is blocked in the case of methyl imidazole and is greater in the case of hexyl imidazole ionic liquid. With the generation of a more open structure, the pollutant will have easier access to it. The experimental data showed a good fit to the Langmuir and Redlich–Peterson isotherms, suggesting that adsorption of the phenol derivative onto the IL@MOF composite does not take place by ideal monolayer sorption, but involves physical sorption. This physical sorption mechanism was also confirmed by the values obtained for the activation energy calculated from the Dubinin–Radushkevich isotherm. Due to this physical adsorption mechanism being needed for the removal of phenol derivatives from aqueous solution, it is important to use adsorbent materials with higher functional groups on the adsorbent surface and a large number of pores and cavities. It was observed that adsorbent materials with the greatest number of active sites available for phenol derivatives could be obtained using a MOF based on a trivalent cation and its functionalization with imidazolium ionic liquid containing a chloride anion and a higher alkyl chain.

## Author contributions

Lavinia Lupa – conceptualization; methodology; validation; visualization; supervision; writing – original draft; writing – review & editing. Nick Tolea: data curation; formal analysis; validation. Marcela Iosivoni – data curation; formal analysis. Bianca Maranescu – data curation; validation; writing – original draft. Nicolata Plesu – investigation; writing – original draft. Aurelia Visa – conceptualization; project administration; resources; supervision; writing – original draft; writing – review & editing.

## Conflicts of interest

The authors declare no conflicts of interest.

## Supplementary Material
